# The Impact of Invariant NKT Cells in Sterile Inflammation: The Possible Contribution of the Alarmin/Cytokine IL-33

**DOI:** 10.3389/fimmu.2018.02308

**Published:** 2018-10-15

**Authors:** Maroua Haroun Ferhat, Aurélie Robin, Louise Barbier, Antoine Thierry, Jean-Marc Gombert, Alice Barbarin, André Herbelin

**Affiliations:** ^1^INSERM U1082 – IRATI Group, Poitiers, France; ^2^Service de Chirurgie Digestive, Oncologique, Endocrinienne et Transplantation Hépatique, CHU Trousseau, Université de Tours, Tours, France; ^3^Service de Néphrologie, Hémodialyse et Transplantation Rénale, CHU de Poitiers, Poitiers, France; ^4^Service d'Immunologie et d'Inflammation, CHU de Poitiers, Poitiers, France

**Keywords:** iNKT, sterile inflammatory response, alarmin IL-33, ischemia reperfusion, CD1d-restricted T cells, tissue repair

## Abstract

Although the contribution of iNKT cells to induction of sterile inflammation is now well-established, the nature of the endogenous compounds released early after cellular stress or damage that drive their activation and recruitment remains poorly understood. More precisely, iNKT cells have not been described as being reactive to endogenous non-protein damage-associated molecular-pattern molecules (DAMPs). A second subset of DAMPs, called alarmins, are tissue-derived nuclear proteins, constitutively expressed at high levels in epithelial barrier tissues and endothelial barriers. These potent immunostimulants, immediately released after tissue damage, include the alarmin IL-33. This factor has aroused interest due to its singular action as an alarmin during infectious, allergic responses and acute tissue injury, and as a cytokine, contributing to the latter resolutive/repair phase of sterile inflammation. IL-33 targets iNKT cells, inducing their recruitment in an inflammatory state, and amplifying their regulatory and effector functions. In the present review, we introduce the new concept of a biological axis of iNKT cells and IL-33, involved in alerting and controlling the immune cells in experimental models of sterile inflammation. This review will focus on acute organ injury models, especially ischemia-reperfusion injury, in the kidneys, liver and lungs, where iNKT cells and IL-33 have been presumed to mediate and/or control the injury mechanisms, and their potential relevance in human pathophysiology.

## Sterile inflammation

Inflammation is an important biological process that represents a coordinated response of the innate immune system against specific molecular patterns present in pathogens or in the damaged tissues of the host. From protozoans to metazoans, the innate immune system, arising about ~1,000 million years ago (1), mediates inflammation as a physiological response to insult, infection, and biological stress in order to restore cellular/tissue integrity, maintain homeostasis and ensure host survival. In recent decades, significant advances have been made on endogenous non-pathogenic activators that trigger inflammation. Endogenous initiators of inflammation can act through the same receptors as pathogens and are referred to as damage-associated molecular patterns (DAMPs). During tissue injury, necrotic cells in response to stress signals (unfolded protein response, oxidative stress response or autophagy) release sterile stimuli, such as DAMPs and alarmins. Hence, in the absence of infection or any pathogenic trigger, sterile stimuli induce the recruitment of inflammatory cells, production of cytokines and chemokines, and induction of T cell-mediated adaptive immune responses (2). This phenomenon is the so-called sterile inflammation response, which uses specific or common pathways to recognize pathogens. Indeed, some DAMPs and alarmins activate pathogen recognition receptors (PRRs) such as Toll-like receptors (TLRs) and the NLRP-3 inflammasome (3, 4), while other endogenous alarmins, such as interleukin (IL)-33, high-mobility group box 1 (HMGB1), and IL-1-α, signal directly *via* specific receptors that are not PRRs (4). Sterile inflammatory response is the initial step toward wound repair mechanisms mediated by macrophages that clear apoptotic neutrophils and produce factors enhancing the resolution of inflammation and the restoration of homeostasis. However, if not resolved, sterile inflammatory responses become pathological (3, 5, 6).

Sterile inflammation is initiated by mechanical, chemical, or metabolic *stimuli*. It occurs in acute conditions, such as ischemia reperfusion injury (IRI), crystal-induced arthritis, trauma, toxin exposure, labor, and with chronic illnesses, such as particle-induced lung diseases and atherosclerosis (3). The identification of the cellular factors and mechanisms of sterile inflammation represents a major issue in the elaboration of efficient therapeutic strategies in human diseases.

## iNKT cells in sterile inflammation: from concept to *in vivo* completion

### Concept

Invariant NKT (iNKT) cells, generally recognized as the archetypal cell subset of innate T-cell receptor (TCR)-αβ lymphocytes, are activated during an early stage of inflammation and subsequently contribute to the development and regulation of innate and adaptive immune responses during infection. However, a major feature of iNKT cells is that their activation does not require the recognition of foreign antigens. Indeed, CD1d-restricted presentation of self-antigens to iNKT cells is induced by endogenous stress and may be stimulated by cytokines that are produced by activated dendritic cells (DCs). Depending on the mode of stimulation, activated iNKT cells rapidly secrete either T helper (Th)1 and Th17 cytokines, interferon (IFN)-γ and IL-17A, respectively, to promote inflammatory responses, or Th2 cytokines, IL-4 and IL-10, to enable repair. iNKT cells therefore represent a unique cell population that is able to sense, trigger and resolve sterile inflammation.

### iNKT cells in the initiation of sterile inflammation: the IRI model

IRI represents a complex inflammatory immune response that generally occurs in a sterile environment and results in tissue damage. IRI has been well-documented in different animal models and in different organs, including kidneys, liver, lungs, heart, and brain.

Furthermore, iNKT cells contribute to early events induced by IRI in different organs including the kidneys (7, 8), liver (9–12), and lungs (13). In brain and heart, iNKT cell recruitment corroborates the severity of IRI, suggesting their implication in the inflammatory response (14, 15).

**Figure 1:**
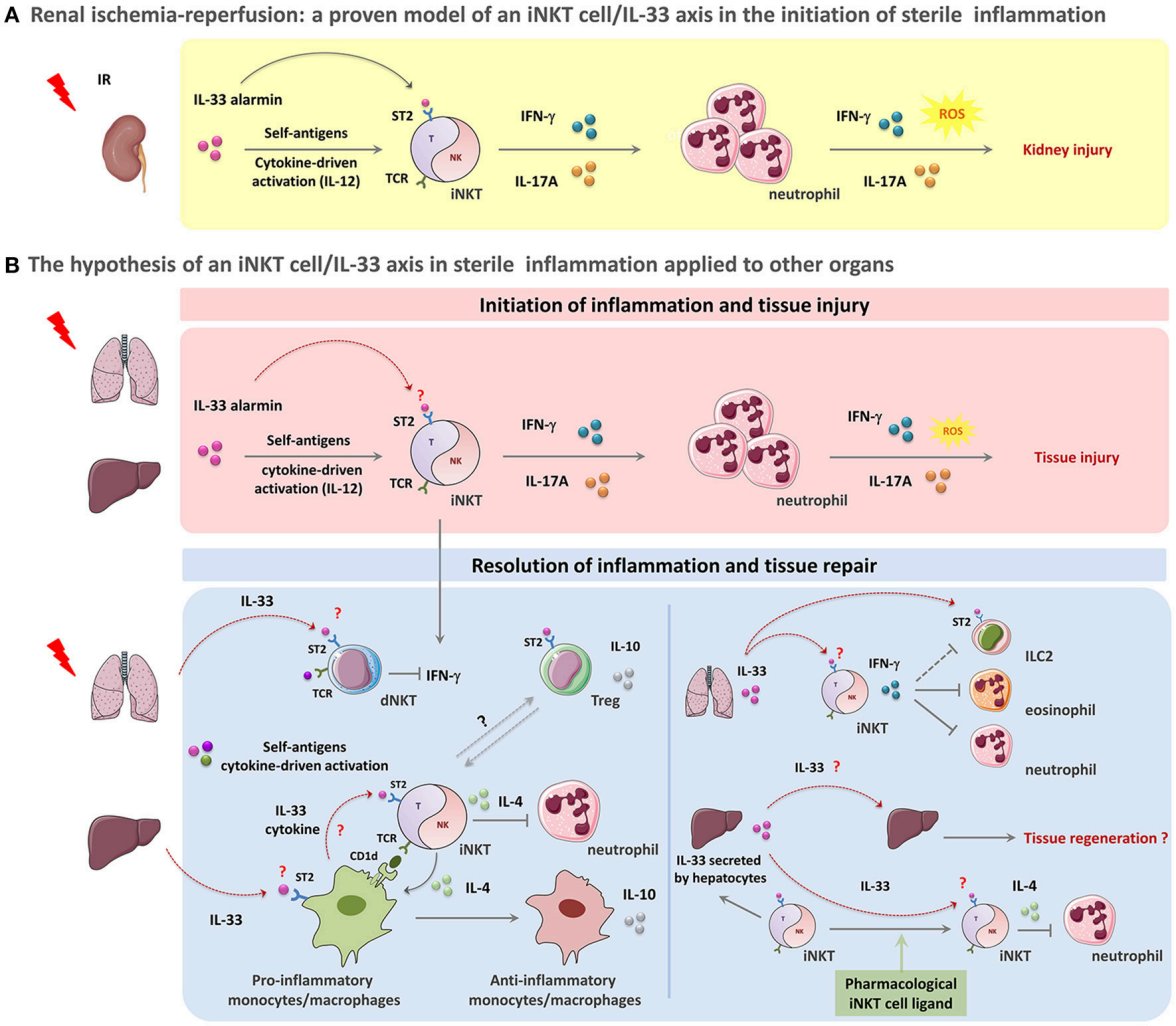
The paradigm of the iNKT cell/IL-33 biological axis in orchestration of acute sterile inflammation. A schematic overview of the potential involvement of iNKT cells in concert with IL-33 in sterile tissue damage **(A,B, upper panel)**) and repair **(B, lower panel)**. **(A,B, upper panel)**: Acute sterile organ injury leads to early iNKT cell activation through the passive release of the alarmin IL-33 that binds to the ST2 receptor constitutively expressed by iNKT cells. IL-33 acts as a requisite co-player leading to complete activation and recruitment of these cells. This mechanism could also involve IL-12 and CD1d-dependent presentation of self-ligands. During kidney IRI, IL-33 released by injured cells promotes iNKT cell activation, recruitment and pro-inflammatory cytokine (IFN-γ, IL-17A) production. IFN-γ- and IL-17A- expressing iNKT cells then contribute to the initiation of inflammation by amplifying neutrophil recruitment, and promoting their pro-inflammatory cytokine and reactive oxygen species (ROS) production, thereby resulting in tissue damage **(A)**. This scenario can be applied to other organs such as liver and lung where both of iNKT cells and IL-33 have been shown separately to contribute to IRI or drug-induced organ injury (for details, see Table 1) **(B, upper panel)**. **(B, lower panel)**: Following acute sterile injury, the innate immune system initiates resolution of inflammation and tissue repair by inducing a shift from M1 to M2 macrophages. By their ability to express Th2-type cytokines, iNKT cells likely contribute to this shift. IL-33 might promote this phenomenon both by recruiting monocytes/macrophages expressing CD1d that in turn activate iNKT cells through their TCR engagement and by amplifying subsequent iNKT cell cytokine production. In this context, activated iNKT cells produce large amounts IL-4, but no IFN-γ. This critical step is a requisite for the transition of monocytes/macrophages from a pro-inflammatory to an anti-inflammatory phenotype with IL-10 production. Th2-type cytokines produced by iNKT cells and monocytes/macrophages suppress inflammation and promote PMN apoptosis **(B, lower left panel)**. Furthermore, IL-33 released shortly after injury could target dNKT (type II NKT) cells and regulatory T cells (Treg), that express ST2 receptor, to counteract IFN-γ-expressing iNKT cells and promote immuno-regulatory cytokine production contributing to the resolution of inflammation **(B, lower left panel)**. In the particular case of lung injury, IL-33 promotes the recruitment of iNKT cells and their IFN-γ production. IFN-γ-expressing iNKT could in turn help to resolve inflammation by counteracting ILC2, eonisophils and neutrophils **(B, lower right panel)**. In the liver, IL-33 can also act as an amplifying factor for ligand-activated iNKT cells, thereby contributing to a shift from the initial pro-inflammatory (pro-Th1) profile of iNKT cells into their (pro-Th2) resolutive profile. As a complementary mechanism, IL-33-driven iNKT cells may in turn sustain protective functions mediated by IL-33 in lung and liver due to their capacity to induce subsequent continuous neosynthesis and secretion of IL-33 by alveolar macrophages and hepatocytes, respectively **(B, lower right panel)**. More precisely, in the liver, neosynthetized IL-33 may promote IL-4-producing iNKT cells that are implicated in the resolution of several sterile inflammatory responses, by suppressing PMN infiltration and enhancing hepatocyte proliferation, thereby preserving tissue function. In this scenario, IL-33 can also directly act on hepatocytes to elicit regeneration after tissue damage **(B, lower right panel)**.

As a common feature, in all of these organs, IRI induces early iNKT cell activation and pro-inflammatory cytokine production, thereby sensing and relaying sterile danger. In the first 24 h following reperfusion, IFN-γ-, Tumor Necrosis Factor (TNF)-α- and IL-17A- producing iNKT cells are closely associated with polymorphonuclear leukocyte (PMN) infiltration and tissue damage. Results have suggested that, once activated, iNKT lymphocytes play a key role in the early development and initiation of sterile inflammation, mainly by rapidly producing large amounts of cytokines contributing to PMN recruitment. Indeed, the use of NK1.1-depleting antibodies, iNKT cell-deficient mice (Jα18 KO or CD1d KO) or reconstitution of iNKT cells by transfer experiments have definitively confirmed the role of iNKT cells in the initiation of IRI responses in kidney (7, 8) (Table 1, Figure 1A), liver (9, 11, 12, 16, 17) and lung (13) (Table 1, Figure 1B, upper panel). Taken together, these studies lead to the conclusion that activation of iNKT cells is a general mechanism for the initiation of IRI. However, the possible involvement of other cell types such as TCR-γδ cells (34–36) and NK cells (37), and their possible interactions with iNKT cells during IRI remain to be explored.

**Table 1:** An overview in mouse of the contribution of the iNKT cell/IL-33 biological axis during acute sterile inflammation.

**Reference(s)**	**Model**	**Organ**	**Role of iNKT cells**	**Mode of action of iNKT cells**	**Role of IL-33**	**iNKT cell/IL-33 axis**
Ferhat et al. (7) Li et al. (8)	IRI	Kidney	initiation (7, 8) Jα18 KO mice	IFN-γ, IL-17 CD1d-dependent	Initiation (7) IL-33 KO mice ST2 KO mice	IL-33-dependent iNKT cell recruitment/activation (7)
Lappas et al. (11) Arrenberg et al. (12) Richards et al. (16) Kuboki et al. (17) Yazdani et al. (18)	IRI	Liver	initiation (11, 12, 16, 17) NK1.1 depletion iNKT-cell adoptive transfer	IFN-γ, IL-17 CD1d-dependent	Initiation (18) IL-33 KO mice	Presumably yes
Arshad et al. (19) Arshad et al. (20)	ConA-induced injury	Liver	initiation (19, 20) iNKT-cell adoptive transfer	IL-4, IFN-γ	Resolution (20)	iNKT-cell-dependent regulation of IL-33 expression by hepatocytes
Cao et al. (21)	IRI	Liver	resolution (21) by iNKT-cell-targeted therapy (α-GalCer)	IL-13 A2AR	Unknown	Unpredictable
Lappas et al. (11)	IRI	Liver	resolution (11) by iNKT-cell-targeted therapy (A2AR agonist)	IFN-γ ↓	unknown	Presumably not
Cheng et al. (22) Antunes et al. (23)	Drug-induced injury	Liver	initiation (22) CD1d KO mice	Not done	Initiation (23) ST2 KO mice	Presumably yes
Liew et al. (24)	Thermal injury	Liver	resolution (24) iNKT-cell adoptive transfer	IL-4	Unknown	Unpredictable
Sharma et al. (13, 25)	IRI	Lung	initiation (13, 25) Jα18 KO mice iNKT-cell adoptive transfer	NOX-2 IL-17	Unknown	Unpredictable
Sharma et al. (25)	IRI	Lung	resolution (25) by iNKT-cell-targeted therapy (A2AR agonist)	NOX-2 ↓ ê IL-17 ↓	Unknown	Unpredictable
Grabarz et al. (26) Kim et al. (27) Bourgeois et al. (28) Li et al. (29) Luzina et al. (30)	Bleomycin-induced fibrosis	Lung	resolution (26, 27)	IFN-γ	Initiation (29, 30)	Presumably yes (28)
Michaudel et al. (31) Pichavant et al. (32) Kubala et al. (33)	Ozone-induced fibrosis	Lung	initiation (32) Jα18 KO mice	IL-17	Initiation (31, 33) resolution (31) IL-33 KO mice	Presumably yes (32) presumably not

*This table recapitulates the main models of acute sterile inflammation, especially IRI models, that involve iNKT cells in different target organs (kidney, liver, and lung). In parallel, when documented, the involvement of IL-33 is indicated; otherwise it is noted as unknown. The implication(s) of iNKT cells and/or IL-33 has (have) been classified according to their contribution to the initiation and/or resolution phase(s) of sterile inflammation, and accompanied by the relevant bibliography. The mode(s) of action of iNKT cells is (are) specified. In the last column, using the cited bibliography, the existence of an iNKT cell/IL-33 axis is shown, surmised (noted ≪ presumably yes ≫) not surmised (noted ≪ presumably not ≫) or difficult to predict (noted ≪ unpredictable ≫). IRI, Ischemia-Reperfusion injury; A2AR, adenosine A2A receptor; α-GalCer, α-galactosylceramide; iNKT, invariant Natural Killer T; KO, transgenic knockout; IFN, interferon; IL, interleukin; NOX-2, NADPH oxidase 2; ↓, decrease*.

## iNKT cells in the initiation/propagation of sterile inflammation: mechanisms of action

### Involvement of stress-induced-endogenous, self-antigen presentation and cytokine-driven signals

The modes of activation and recruitment of iNKT cells in induction of sterile inflammation remain poorly understood. As innate immune system components, iNKT cells are expected to rapidly and efficiently respond to cell-stress and represent an obvious candidate to participate in endogenous pathways of inflammation, especially in sterile inflammation. However, iNKT cells have not been described as reactive to endogenous non-protein DAMPs that include ATP, uric acid, heparin sulfate and DNA. Moreover, although iNKT cells express the adenosine 2A receptor (A2AR), their reactivity to ligands of this receptor has been described only under pharmacological conditions and has resulted in a downregulation rather than an induction of iNKT cell functions (11, 25) (Table 1).

Numerous studies have documented the involvement of TCR/CD1d interactions in iNKT cell activation during sterile inflammation induced by IRI. In most cases, the blockade of CD1d presentation with anti-CD1d antibodies has prevented iNKT cell-mediated renal and hepatic IRI (8, 11, 16, 17). However, further investigations are needed to characterize the endogenous lipids presented by CD1d and to identify the effective presenters of CD1d-relevant lipids. It also remains to determine the inducible role of non-protein DAMPs in their expression by CD1d-presenting APCs.

A specific feature of iNKT cells is their ability to be activated by cytokine-driven signals independently of TCR-engagement and CD1d recognition. During sterile inflammation, their innate IFN-γ producing capacity does not require continuous auto-antigen recognition in mice (38, 39), corroborating the *in vitro* demonstration that the pro-inflammatory cytokine IL-12 (alone or in combination with IL-18) can activate iNKT cells to produce IFN-γ. Indeed, IL-12 and IL-18 amplify both Th1- and Th2-like iNKT cell responses upon TCR engagement (40–43). Accordingly, during renal IRI, we have documented an increase of plasma IL-12, while Marques et al. (44) have reported protection of IL-12-deficient mice. Moreover, in a model of sterile liver injury, Liew et al. (24) highlighted a biphasic mechanism of iNKT cell activation through self-antigen presentation and IL-12/IL-18-driven signals. Lastly, during experimental cerebral ischemia, where iNKT cells have been reported to accelerate brain infarction (14), early detrimental T-cell effects have not been associated with adaptive immunity (36). Taken together, these results from the literature demonstrate that iNKT cells mediate acute sterile inflammation, including IRI, through TCR-engagement and cytokine-driven signals (Table 1; Figure 1A; Figure 1B, upper panel).

### A key role for the alarmin IL-33 in iNKT cell activation and recruitment in sterile inflammation?

#### The archetypal alarmin/cytokine IL-33

Alarmins, a second subset of DAMPs, are tissue-derived nuclear proteins, constitutively expressed at high levels in epithelial barrier tissues and endothelial barriers. These potent immunostimulants include defensins, cathelicidin, eosinophil-derived neurotoxin, HMGB1, IL-1-α, IL-18, and IL-33. Once released by necrotic cells after tissue damage, they can activate TLRs or cytokine receptors, and serve as early warning signals to alert adjacent cells/tissues and to mobilize innate and adaptive immune systems.

Among alarmins, the newest member of the IL-1 super-family is IL-33, also called IL-1F11. IL-33 has aroused interest due to its singular action during infectious and allergic responses (45, 46), and acute tissue injury (31, 47). Indeed, IL-33 acts as an alarmin released by necrotic cells after tissue damage, and as a cytokine, due to its inducible expression and subsequent continuous secretion by hematopoietic cells like mastocytes and macrophages (31, 45). By interacting with the ST2/IL-1RAcP receptor complex, IL-33 targets adaptive immune cell subsets, namely conventional CD4 and CD8 T cells and regulatory T cells (Treg). IL-33 also targets several innate immune cells including iNKT cells, NK cells, mastocytes, group 2 innate lymphoid cells (ILC2) and myeloid-derived suppressor cells, thereby influencing their functions and homeostasis (42, 48–50). IL-33 is therefore a crucial alarmin and an ubiquitous immune modulator, with a pivotal role in sterile inflammation.

#### The alarmin IL-33 in sterile inflammation: relevance to IRI models

It is generally understood that mechanical, chemical, trauma injury and IRI to several organs, including kidney, liver, lung, and brain, lead to rapid release of IL-33, presumably by damaged cells, further supporting the role of IL-33 as an alarmin (7, 18, 29, 31, 47, 51, 52). However, except during brain trauma (47) and kidney IRI (7) (and section First Evidence of an iNKT Cell/IL-33 Biological Axis Mediating Inflammation During Renal IR: Relevance to Liver and Lung?), it remains to be demonstrated that full-length active IL-33 disappears from the nucleus of damaged cells and immediately increases in circulation. The immune effectors of inflammation and repair targeted by IL-33 also need to be identified. In most of these situations, it is well-recognized that IL-33 release precedes iNKT cell recruitment and/or local activation, opening the question of the role of IL-33 as an early warning signal to alert iNKT cells in sterile inflammation responses.

#### IL-33 targets iNKT cells

The existence of an iNKT cell/IL-33 biological axis is well-established. Indeed, even though IL-33 alone is not able to fully and completely activate iNKT cells, the alarmin can directly target them as an essential amplifying factor in both primary innate and adaptive immune responses. iNKT cells have an immediate biological reactivity to IL-33 because, like NK cells, they constitutively express on their surface the ST2 chain specific of the IL-33 receptor (42, 49). As a result, IL-33 contributes as a co-stimulatory factor to type 1 (IFN-γ), type 2 (IL-4, IL-10), and type 17 (IL-17A) iNKT cell cytokine production profiles upon TCR engagement (7, 42). Moreover, in combination with IL-12, IL-33 enhances IFN-γ production by iNKT cells. Along with recruitment and local activation of iNKT cells, these functions depend on endogenous IL-12 (28, 42). The same demonstration has been documented in other mammals such as humans (49, 53) and pigs (54). Taken together, these data support the conclusion that IL-33 can recruit iNKT cells and contribute as a co-stimulatory factor to pro-Th1-, pro-Th2-, and pro-Th17- iNKT cell responses in an IL-12-dependent manner.

#### First evidence of an iNKT cell/IL-33 biological axis mediating inflammation during renal IR: relevance to liver and lung?

Given that both iNKT cells and IL-33 have been shown to contribute to acute organ injury in both kidneys and liver, IRI seems to represent a suitable model of sterile inflammation to test the physiological significance of the iNKT cell/IL-33 biological axis. Therefore, we recently provided the first demonstration that endogenous IL-33 contributes as an alarmin to IRI in the kidneys. Indeed, we highlighted IL-33 rapid release (≤1 h) from its constitutively full-length form expressed at high levels in the nuclei of kidney cells and its transient presence in the extracellular space. Moreover, we further characterize a previously undefined mechanism where IL-33/ST2 engagement promotes iNKT cell recruitment, IFN-γ and IL-17A cytokine production, in the presence of IL-12 as a co-factor for IL-33, resulting in PMN infiltration and activation (7) (Figure 1A).

We presume that the coordinated action of IL-33 and iNKT cells will also apply to the liver, where they have been separately shown to contribute to IRI, and where, as in the kidneys, IRI severity depends on IFN-γ and IL-17A (11, 18) (Figure 1B, upper panel). Regarding lung IRI, an exacerbating role has been attributed to IL-17A-producing iNKT cells, but in this model, IL-33 release as an alarmin to amplify IL-17A expression by iNKT cells has not yet been addressed (Figure 1B, upper panel).

#### The iNKT cell/IL-33 biological axis: a paradigm extended to ozone- and drug- induced organ injury

The iNKT cell/IL-33 biological axis deserves particular attention when considering organ injury caused by chemical components such as ozone or drugs (Table 1, Figure 1B, upper panel). Indeed, ozone-driven lung inflammation that requires the presence of iNKT cells (32) is associated with IL-33 release by epithelial cells (33). Furthermore, iNKT cells have been shown to be involved in halothane-induced liver injury (22), while blockade of the IL-33/ST2 axis reduces acetaminophen-mediated organ injury (23). It remains to determine whether iNKT cells and IL-33 act in a concerted manner to initiate sterile inflammation in response to liver-targeted drugs.

## The hypothesis of a functional axis between iNKT cells and IL-33 in the resolution of sterile inflammation

### Impact of iNKT cells on the resolution of sterile inflammation

Inflammation is a physiopathological and protective response of the organism (host) to infection or sterile tissue damage aimed at neutralizing and eliminating the causing agent/insult. Shortly after the beginning of the inflammatory response, a coordinated and active resolution program involving PMN apoptosis and clearance initiates in order to restore tissue integrity and organ function (6). The resolution of acute inflammation is crucial to ensure proper return to homeostasis and to avoid persistent chronic inflammation, including metabolic diseases and autoimmune syndromes.

Innate immune cells are key actors that orchestrate the switch from acute inflammation to resolution. As regards iNKT cells, one may presume that their functional malleability renders them capable of intervening not only in initiation but also in the resolution of sterile inflammation. However, few studies have described the role of iNKT cells in the resolution of sterile inflammation (24, 26, 27, 55) (Table 1). For example, in experimental models of acute liver injury (IRI (9, 10) and ConcanavalinA (ConA)-induced hepatitis (56, 57), iNKT cells display a pro-inflammatory deleterious phenotype. A resolutive role attributed to iNKT cells has only been documented in a drug-induced injury model (58), where iNKT cells have been shown to orchestrate a switch from inflammation to resolution of sterile injury. In response to thermal trauma in the liver, another underlying mechanism of resolution is iNKT cell-derived IL-4, which drives the shift from M1 to protective M2 macrophages (Figure 1B, lower left panel). Interestingly, this mechanism is similar to a reported model of sterile inflammation in the peritoneum (55). However, IFN-γ-producing iNKT cells, rather than their IL-4-producing counterparts, are resolving in several models of sterile inflammation in the lung (26–28) (Table 1). Taken together, these data reveal that iNKT cell functions required to resolve sterile inflammation and to promote tissue repair strongly depend on the organ microenvironment.

In addition to their natural resolutive function, iNKT cells can orchestrate sterile inflammation in the liver when targeted by their pharmalogical ligand α-Galactosyl-Ceramide (α-GalCer). This pharmacological ligand appears to act by shifting the initial pro-inflammatory (pro-Th1) profile of iNKT cells into their resolutive (pro-Th2) profile (21) (Table 1).

Other evidence suggests that iNKT cells and type II NKT cells (also called dNKT cells due to their expression of oligoclonal TCRs and recognition of self-antigens, including sulfatide, in a CD1d-dependent manner) have opposing roles at an early stage of liver inflammation (59). Further studies are needed to determinate if the interactions between the two NKT cell-subsets constitute a general mechanism coordinating initiation and resolution of sterile inflammation. Indeed, it was recently reported that pharmacological activation of dNKT cells by the self-glycolipid antigen sulfatide led to reduced IFN-γ secretion by iNKT cells and prevented hepatic and renal IRI (12, 60) (Figure 1B, lower left panel).

Up until now, without pharmacological intervention, there has been no reported experimental model in which iNKT cells exercise their initiating and resolution functions in a coordinated manner. Indeed, the highlighting of two opposite functions of the same cell type in a given experimental model is a challenge. Furthermore, in most IRI models, iNKT cell-dependent tissue lesions are severe and render the resolution phase difficult to analyze.

### Are iNKT cells involved in concert with IL-33 to promote resolution and repair in sterile inflammation?

Due to its ability to induce epithelial and endothelial cell proliferation (48, 61), the involvement of IL-33 as a factor promoting repair and tissue regeneration following stress has been proposed. Another element supporting the role of IL-33 in tissue repair is the targeting of Treg and ILC2, contributing to resolution of inflammation of the intestine (62), skeletal muscles (63) and skin/wound repair (64). Lastly, IL-33 counteracts pro-Th1 inflammatory responses by targeting the shift from M1 to M2 macrophages (51, 65, 66). Whether these protective IL-33-driven functions are influenced by iNKT cells is unknown because interactions between Treg and iNKT cells, or ILC2 and iNKT cells, have been described only under pharmacological intervention (67, 68).

However, the iNKT cell/IL-33 biological axis can function as an additional mechanism in several situations, like in the peritoneum and liver, where an IL-4-dependent macrophage-iNKT cell circuit suppresses the sterile inflammation response (24, 55). As IL-33 can dramatically increase both IFN-γ and IL-4 productions by iNKT cells, it is unclear whether in this context it favors iNKT cell-mediated protection. We presume that this will occur at least during thermal-induced liver injury where iNKT cells produce IL-4 rather than IFN-γ (24) (Figure 1B, lower left panel).

In the lungs, IL-33 itself may be crucial for resolution of tissue injury by amplifying IFN-γ expression by iNKT cells (Figure 1B, lower right panel). Indeed, when administered at pharmacological doses, IL-33 induces ILC2-mediated airway inflammation (69) and controls this response *via* a mechanism that involves IFN-γ-expressing iNKT cells (28). From these data, we propose that IL-33 not only targets ILC2 to promote pro-Th2 inflammatory response, but also contributes concomitantly to recruit iNKT cells and activate their production of IFN-γ. In this way, IL-33 acts as a negative feedback loop to resolve lung inflammation, possibly by counteracting neutrophils (70) and/or ILC2 (71). Interestingly, in response to bleomycine, IL-33 potentiates lung injury (29, 30) whereas iNKT cells attenuate the deleterious response by down modulating the Th2 milieu (26) and producing IFN-γ (27). Taken together, these findings favor the hypothesis that IL-33-driven IFN-γ-expressing iNKT cells may represent a natural mechanism of resolution during sterile inflammation in lung (Figure 1B, lower right panel).

It should be emphasized that iNKT cells may also contribute in an indirect manner to protective responses driven by IL-33, by amplifying the inducible synthesis of the alarmin/cytokine (see Figure 1B, lower right panel). Indeed, iNKT cells themselves contribute to the subsequent transcriptional and protein synthesis of IL-33 by alveolar macrophages and hepatocytes during acute sterile inflammation responses in lung and liver, respectively (19, 69). Thus, in the above-mentioned ozone-induced lung inflammation model where iNKT cells are requisite (32), a biphasic injury and inflammation controlled by IL-33 has been proposed (31), as previously shown in an anti-parasitic response model (45). IL-33 is first released as an alarmin by epithelial cells and then its synthesis is relayed by alveolar macrophages (*vs*. mast cells in the anti-parasitic model), thereby triggering tissue protection/repair (*vs*. boosting a pro-Th2 protective antiparasitic response). It would be important to determine whether iNKT cells contribute to this biphasic function of IL-33. Lastly, during acute hepatic injury induced by ConA, iNKT cell-dependent IL-33 synthesis occurs precisely in hepatocytes (19). As IL-33 is recognized as hepatoprotective (20), one may hypothesize that iNKT cells, by increasing the synthesis of IL-33 in hepatocytes, contribute to the repair phase of hepatocyte damage (Figure 1B, lower right panel). This hypothesis can also be applied to hepatic IRI, where IL-33 synthesis is induced in hepatocytes from 4 h after reperfusion (18).

## Therapeutic strategies targeting the iNKT cell/IL-33 biological axis

While recent studies have highlighted the pathogenic mechanisms of iNKT cell responses, the beneficial functions of iNKT cells are just beginning to be explored. Pharmacological and cell-based therapies influencing iNKT cell responses in experimental acute organ injury suggest that this is a promising approach for the preservation of organ function during sterile inflammation. We surmised that this approach should take into account the potential implication of IL-33.

Activation of iNKT cells by their pharmacological ligands during sterile inflammation, especially IR, can be protective or exacerbating, depending on the state of APCs (72) and the organ involved (14, 15, 21). IL-33 works as an amplifying factor for ligand-activated iNKT cells (7, 42) and as a protective factor by targeting Treg and ILC2 (50) or parenchymal cells like hepatocytes (73) and myocardiocytes (74, 75). As a result, it can be argued that protection driven by α-GalCer would be more robust if the iNKT cell ligand was co-administered with IL-33. Indeed, in liver and heart IR models, the delivery of either α-GalCer (74, 75) or exogenous IL-33 alone (15, 21) has shown protective effects (Table 1; Figure 1B, lower right panel). In this context, the contribution of IL-33 to the α-GalCer-driven iNKT cell/Treg cross-talk (67, 68) deserves particular attention.

Treatment with IL-33 alone would also be beneficial when sterile inflammation is accompanied by the natural protective effects of iNKT cells (24, 55). Conversely, in kidney IR models where initiation of inflammation depends on the iNKT cell/IL-33 biological axis, IRI would be counteracted by early blockade of IL-33 together with agonist A2AR treatment, known to attenuate IFN-γ-expressing iNKT cell activation (11, 25).

Taken as a whole, even though therapeutic strategies targeting the iNKT cell/IL-33 biological axis are promising, they might be complex to develop, given that the contribution of iNKT cells and IL-33 in inflammation to exacerbated illness *vs*. improved recovery, largely depends on the model.

## Concluding remarks

Focusing on recent advances in the understanding of the biology of iNKT cells and the alarmin IL-33 obtained from animal models, we propose that iNKT cells and IL-33 form a biological axis in alerting and controlling the immune cells involved in sterile inflammation associated with tissue damage.

The review shows that the involvement and the underlying mechanisms of the iNKT cell/IL-33 biological axis in sterile inflammation depend on the model, organ microenvironment, and the initiating *vs*. resolutive/repair phase of the inflammation response. Even though more work is required in this area, this review brings new evidence that the iNKT cell/IL-33 biological axis acts during sterile inflammation induced by IR in kidneys and liver and after chemical- or drug- induced acute tissue injury in the kidneys, liver and lungs.

At this stage, an important challenge is to determine how much of the current information in animal models can accurately be translated to human patients. Given the widely recognized deleterious action of the iNKT cell/IL-33 biological axis in renal IR in mice, an immediate challenge would be to improve understanding of the physiopathological impact of this biological axis during IR sequences in organ transplantation. As far as kidney transplantation is concerned, a pilot clinical study indicated a prompt release of lL-33 into the circulation early after organ reperfusion that could be responsible for the activation of iNKT cells (53). Moreover, IL-33 levels and IRI duration are correlated, supporting a close connection between kidney cell injury, IL-33 release and iNKT cell activation. That iNKT cells and IL-33 may also function in a coordinated manner after liver transplantation in humans is an attractive hypothesis and deserves further investigation in our opinion.

*In fine*, new therapeutic strategies targeting the iNKT cell/IL-33 biological axis could prove beneficial for the long-term survival of organs after acute organ injury. If so, the secondary “repair” functions of the iNKT/IL-33 biological axis, once identified, would need to be protected from treatment focused on the initial, deleterious phase.

More generally, exploration of the crucial and diverse roles of the iNKT cell/IL-33 biological axis during acute sterile inflammation settings may contribute to understanding the mechanisms that control the switch between healthy and pathological inflammation.

## Author contributions

LB, AT and J-MG contributed to literature search for the review. AB and AR contributed to literature search and writing for the review. MF and AH contributed to the literature search for the review, provided writing, and editing of the review.

### Conflict of interest statement

The authors declare that the research was conducted in the absence of any commercial or financial relationships that could be construed as a potential conflict of interest.
